# Rhinosporidiosis: A Rare Cause of Proptosis and an Imaging Dilemma for Sinonasal Masses

**DOI:** 10.1155/2016/3573512

**Published:** 2016-11-30

**Authors:** Amit Kumar Dey, Rajaram Sharma, Kartik Mittal, Puneeth Kumar, Vivek Murumkar, Sumit Mitkar, Priya Hira

**Affiliations:** Department of Radiology, King Edward Memorial Hospital and Seth G.S. Medical College, Room No. 107, KEM Main Boy's Hostel, Parel, Mumbai 400012, India

## Abstract

*Background.* Rhinosporidiosis is a common disease entity in tropical countries; however, it can be encountered in other parts of the world as well due to increasing medical tourism. It may mimic other more malignant and vigorous pathologies of the involved part.* Case Report.* We present a case of a 36-year-old male presenting with proptosis due to involvement of nasolacrimal duct which is rare. We will discuss typical CT and MRI features of the disease which were present in the case.* Conclusion.* For a surgeon and a radiologist, this is a necessary differential to be kept in mind for sinonasal masses. CT and MRI are invaluable investigations. However, FNAC is confirmatory. Both clinical and radiological aspects are required to reach correct diagnosis.

## 1. Introduction

Rhinosporidiosis is mainly prevalent in south Asian countries. Frequent bathing in stagnant water ponds is a risk factor for this infection [1]. It is a chronic granulomatous disease caused by the fungus* Rhinosporidium seeberi*. Pathologically, there is nasal polyposis and other manifestations like hyperplasia of nasal mucosa [2]. Recurrence is quite common after surgical excision [3]. This is primarily a disease of orofacial region. In decreasing frequency, it involves nasal cavity, nasopharynx, oropharynx, and nasolacrimal duct. However, other viscera, trachea, bones, brains, and orbits have also been involved [4].

Here, we are presenting a case of a 36-year-old male patient presenting with proptosis due to involvement of nasolacrimal duct which is rare. We will discuss typical CT and MRI features of the disease which were present in the case.

## 2. Case Report

A 36-year-old male presented with long standing history of proptosis of right eye since 1 year and foul smelling nasal discharge since 2 months. The patient also gave history of spells of nasal itching. There is no history of constitutional symptoms. There is no history of similar complaints in the family. He works as a farmer in rice fields. Local examination of the eye showed proptosis of lower eyelid and there was a firm and round swelling near medial canthus of right eye. Local examination of the nasal cavity showed friable polypoidal reddish mass in inferior turbinate which was extending into opening of nasolacrimal duct.

In nasopharynx multiple polypoidal reddish friable mass which would bleed on touching was seen originating from the base of the skull. Biochemical and haematological tests done on the patient were normal.

A CT scan of paranasal sinuses and orbits was ordered which showed intensely enhancing extraconal mass lesion in inferior portion of right eye. There was rarefaction of inferior orbital wall and bony portion of nasolacrimal duct. Nasolacrimal duct was enlarged with rarefaction of bony walls.

**Figure 1:**
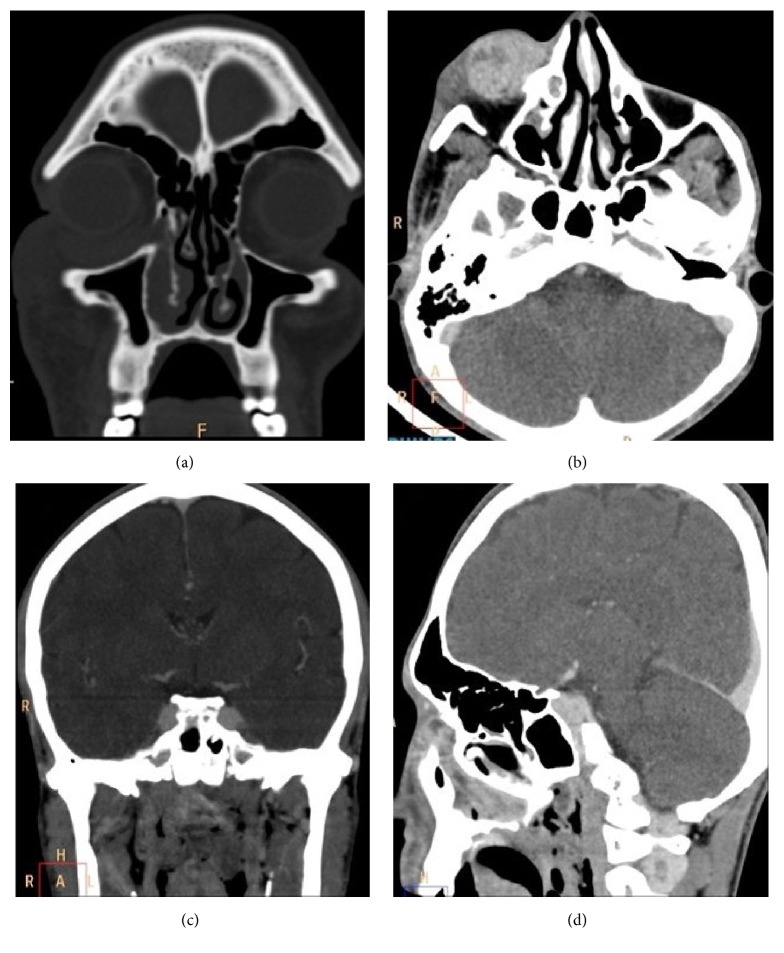
(a) Coronal image of CT paranasal sinuses in bone window showing soft tissue density mass lesion in inferior portion of right eye with extension into nasolacrimal duct. Nasolacrimal duct is expanded with rarefaction of its walls. Mass is reaching up to inferior turbinate. (b) CT postcontrast axial view in soft tissue window at the level of maxillary antrum showing well-defined, intensely enhancing mass lesion anteroinferior to right globe. (c) CT coronal image in maximum intensity projection showing abnormally hypertrophied vessels originating from nasopharyngeal mucosa and supplying the lesion. (d) CT postcontrast sagittal view in soft tissue window showing extension of the lesion through the expanded nasolacrimal duct into inferior turbinate.

**Figure 2:**
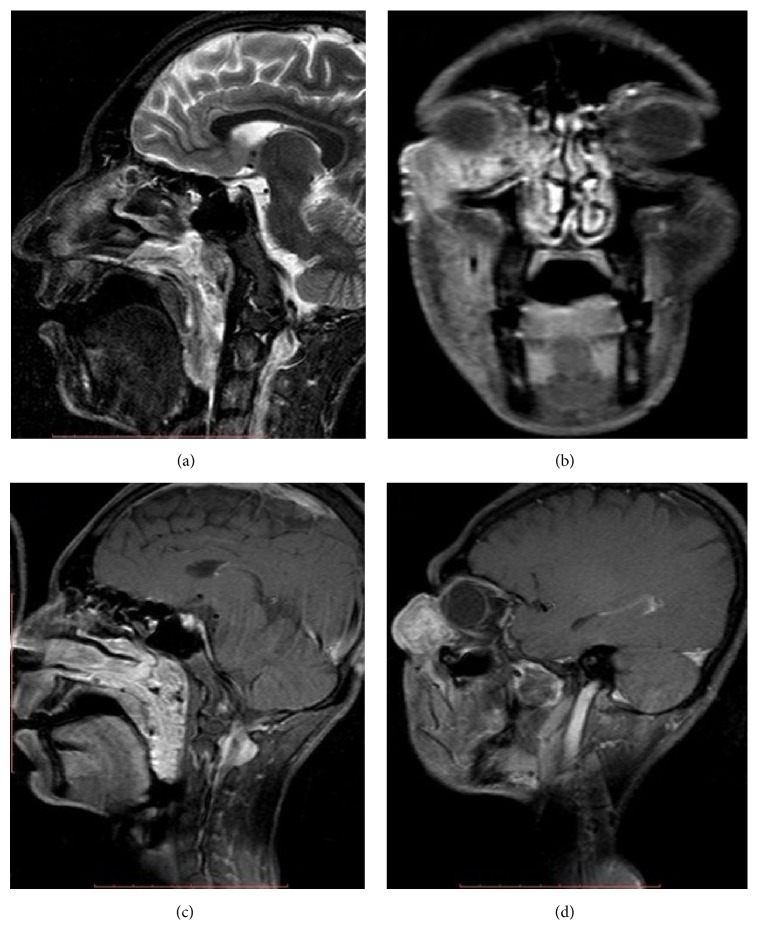
(a) T2-weighted MRI sagittal section showing heterogeneously hyperintense multilobulated mass originating from nasal cavity and nasopharynx and extending into oropharynx. Lesion shows few flow voids within. (b) Postcontrast T1-weighted MRI coronal section showing enhancing mass lesion in inferior portion of right orbit. There are few nonenhancing areas within. Lesion is extending into the inferior turbinate via nasolacrimal duct. (c) Postcontrast T1-weighted MRI sagittal section showing intensely enhancing multilobulated mass originating from nasal cavity and nasopharynx and extending into oropharynx. Lesion shows few nonenhancing areas within. Lesion shows multilobulated external surface mimicking cerebriform appearance of inverted papilloma. (d) Postcontrast T1-weighted MRI sagittal section showing extraconal, intraorbital, intensely enhancing mass extending into the nasal cavity via nasolacrimal duct. The mass is protruding outside of the orbital margins causing proptosis.

Similarly, enhancing mass was seen in inferior turbinate and nasopharynx. There were few areas of air specs and calcification within the lesion. There was a leash of blood vessels seen originating from the nasopharyngeal wall and supplying the mass (Figure 1). Following this, MRI was done which showed the following (Figure 2):

- T2-weighted sagittal section showing heterogeneously hyperintense multilobulated mass originating from nasal cavity and nasopharynx and extending into oropharynx. Lesion shows few flow voids within
- Postcontrast T1-weighted coronal section showing enhancing mass lesion in inferior portion of right orbit. There are few nonenhancing areas within. Lesion is extending into the inferior turbinate via nasolacrimal duct
- Postcontrast T1-weighted sagittal section showing intensely enhancing multilobulated mass originating from nasal cavity and nasopharynx and extending into oropharynx. Lesion shows few nonenhancing areas within. Lesion shows multilobulated external surface mimicking cerebriform appearance of inverted papilloma
- Postcontrast T1-weighted sagittal section showing extraconal, intraorbital, intensely enhancing mass extending into the nasal cavity via nasolacrimal duct. The mass is protruding outside of the orbital margins causing proptosis

**Figure 3:**
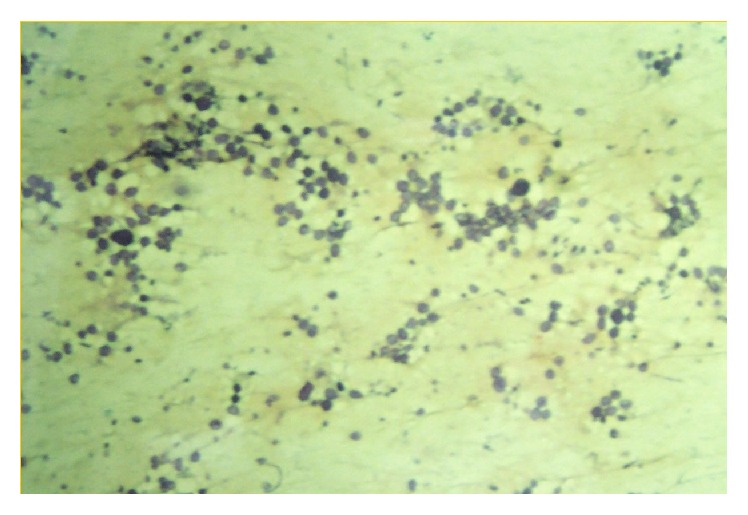
H&E slide prepared by fine-needle aspiration cytology from the lesion showing groups of spore of rhinosporidium of varying sizes.

Finally, FNAC (H&E staining) of the orbital lesion was done, which showed groups of spore of rhinosporidium of varying sizes (Figure 3). Following this, wide excision of the nasolacrimal duct/orbital lesion and electric cauterization of the base of the orbital lesion were done. Endoscopic sinus surgery of the nasopharyngeal lesion was done and to prevent recurrence patient was put on dapsone therapy. The patient did remarkably well and showed no signs of recurrence on follow-up.

## 3. Discussion

Rhinosporidiosis is a chronic granulomatous disease caused by* Rhinosporidium seeberi* presently classified in class Mesomycetozoea [5]. The pathogen enters the host primarily by abraded skin or mucosa via transepithelial spread [6]. This explains nose as being the most common site in involvement of the disease. However, other methods like autoinoculation, haematogenous spread, and direct inoculation have been described for other visceral involvements [7, 8]. Nasal involvement can be a single pedunculated polyp or multiple sessile polyps arising from mucosa [9]. Other manifestations like multiple cutaneous reddish polyps, bone lesions, and corneal mass are described by few case reports. Diagnosis in the cases affecting nose and throat can be done by clinical examination. However, atypical presentation like ours and lower respiratory tract involvement require radiological examination. CT and MRI are also needed for depiction of extent of the lesion and complications and in cases of recurrence to plan surgery.

On CT, it looks like irregular or lobulated lesions of soft tissue density showing moderate-to-intense postcontrast enhancement. Small foci of calcification and air can be seen within the lesions. Multiple dilated vessels can be also seen arising from nasopharyngeal mucosa which can be seen supplying the lesion. Lesions arising from oropharynx or trachea can be relatively hypoenhancing compared to the lesions at other sites. Bony involvement may appear as thinning of wall, rarefaction, or complete erosion [10].

MRI shows heterogeneous mixed density mass lesion with prominent flow voids on T2-weighted imaging. Postcontrast imaging shows intense enhancement of the mass. Multilobulated appearance may give rise to cerebriform appearance as described for inverted papilloma in the literature. The main imaging differentials are inverted papilloma, juvenile angiofibroma, lobular capillary hemangioma, angiomatous polyp, and sinonasal malignancy [10].

Diagnosis can be done by FNAC and examination under 10% KOH or Papanicolaou smear. However, usually histopathological examination is required. Both of the above methods show pathogen in its various stages of development [11, 12].

Surgical removal with wide margins remains the main cornerstone of the treatment with electrocautery of the base. Dapsone can be added as adjuvant medical therapy; however, no definite benefits of dapsone therapy have been proven. Even after the treatment with wide excision, there are high chances of recurrence probably due to hematogenous spread of the disease during surgery [13–15].

## 4. Conclusion 

Rhinosporidiosis is a common disease entity in tropical countries; however, it can be encountered in other parts of the world as trend of medical tourism is increasing. It may mimic other more malignant and vigorous pathologies of the involved part. For a surgeon and for a radiologist, this is a necessary differential to be kept in mind for sinonasal masses. Both clinical and radiological aspects are required to reach correct diagnosis.

Inferior meatus is a common site of involvement in the nasal cavity because of its close proximity with the nasolacrimal duct. It is more likely that lesion began in the nasal cavity and then extended into the orbit via the nasolacrimal duct. The presence of other lesions in the nasopharynx may further support this.
